# Commercially Supplied Amine-Modified siRNAs May Require Ultrafiltration prior to Conjugation with Amine-Reactive Compounds

**DOI:** 10.4061/2011/154609

**Published:** 2011-05-29

**Authors:** Shannen Lau, Bim Graham, Ben J. Boyd, Colin W. Pouton, Paul J. White

**Affiliations:** Monash Institute of Pharmaceutical Sciences, Monash University, 381 Royal Parade, Parkville, VIC 3052, Australia

## Abstract

Conjugation of siRNA to macromolecules such as serum albumin has multiple potential benefits, including enhanced extravasation via albumin-mediated transcytosis across endothelial cells and reduced renal clearance. In attempting to conjugate siRNA to albumin, we used commercially sourced amine-modified siRNA and reacted it with the heterobifunctional linker succinimidyl 4-[*N*-maleimidomethyl]cyclohexane-1-carboxylate (SMCC) to introduce a maleimide group suitable for conjugation to the thiol group of the surface-exposed cysteine residue (Cys 34) within albumin. We found the conjugation of the SMCC-treated siRNA to bovine serum albumin (BSA) to be very inefficient and investigated the cause of the low yield of conjugate. Ultrafiltration with phosphate-buffered saline prior to activation with SMCC dramatically increased the yield of siRNA-albumin conjugate (~15-fold). Communication with the commercial supplier revealed that ammonium acetate buffer was used in a desalting step as part of the siRNA purification process prior to supply, likely resulting in ammonium counterions to the siRNA polyanion, which would interfere with conjugation by consuming the SMCC. After ultrafiltration, a greatly reduced amount of SMCC could be used to affect conjugation, without significant reduction in yield. These data indicate that amine-modified siRNA sourced commercially may require ultrafiltration or dialysis prior to use in conjugation reactions.

## 1. Introduction

A series of obstacles impede the successful delivery of short interfering RNA (siRNA) to the cytosol of target cells [1, 2]. Strategies involving conjugation of siRNA to a wide variety of molecules have been employed to overcome these obstacles. Typically, these approaches employ amine- [3] or thiol-modified [4, 5] siRNA at 3′ or 5′ ends of one strand of the siRNA duplex (see [6] for review of conjugation chemistry). Conjugations of siRNA to polyethylene glycol [5], quantum dots containing targeting moieties [4], cell penetrating peptides such as penetratin [7], and a range of lipophilic molecules, including cholesterol [8], have been demonstrated without a loss of silencing ability. 

**Figure 1:**
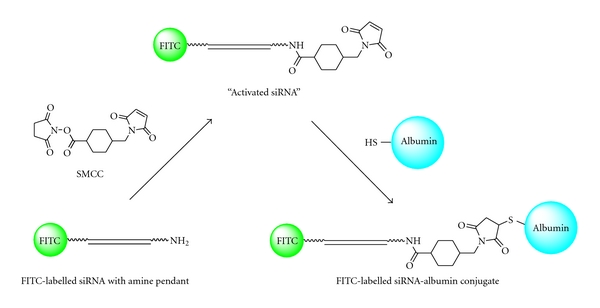
Conjugation chemistry. Formation of an amide bond between the heterobifunctional cross-linker SMCC and commercially sourced amine-modified siRNA produces maleimide-functionalized siRNA (activated siRNA). The activated siRNA reacts with cysteine-34 of BSA to produce an siRNA-BSA conjugate.

Recently, we embarked on a project aiming to deliver siRNA to the heart using siRNA conjugated to the serum protein albumin. The strategy employed involved the use of the common heterobifunctional linker succinimidyl 4-[*N*-maleimidomethyl]cyclohexane-1-carboxylate (SMCC). Using this approach, the coupling of SMCC to siRNA containing an aminohexyl pendant at the 3′ end of the sense strand results in an “activated” siRNA bearing a thiol-reactive maleimide group. The “activated” siRNA is then covalently linked to serum albumin via reaction with the thiol-containing side chain of cysteine residue 34, located on the surface of serum albumin molecules (see Figure 1). Previous studies have shown that a number of thiol-reactive drugs rapidly and selectively bind to endogenous albumin within a few minutes of administration, due to that fact that free thiol groups are not found on the majority of circulating serum proteins except for albumin [9]. The chemistry used to conjugate the siRNA to albumin is not novel; others have conjugated nanoparticles to siRNA using a similar cross-linker, *N*-succinimidyl-3-(2-pyridyldithio)propionate (SPDP) [10]. These workers employed a 40 : 1 SPDP : siRNA ratio to activate the siRNA and then gel filtration to remove the unreacted SPDP, prior to conjugation. We found significant batch-to-batch variability in the efficiency of the siRNA conjugation to bovine serum albumin (BSA) using similar conditions and therefore sought to understand the factors limiting the conjugation. Here, we report a successful strategy to restore conjugation efficiency, by repeated buffer exchange prior to exposing the amine-modified siRNA to SMCC.

## 2. Experimental Methods

### 2.1. Materials

Samples of IGF-IR siRNA used were sourced from Dharmacon (CO, USA) and were modified with an amine for conjugation, with 2′-O-methyl modifications for *in vivo* use, and with a fluorescein label for imaging purposes. Each sense strand contained four 2′-O-methyl modifications, whilst the antisense strand contained two 2′-O-methyl modified nucleotides. The sense strand contained a 3′ amine modification (N6) and a fluorescein label (Fl) at its 5′ end. The sequence used was as follows, where “m” refers to 2′-O-methyl modification:

sense strand: 5′-Fl(*)GCmCmCAUmGUGUGAGAmAGACC(*)dT(*)dT(*)N6-3′,

antisense strand: 3′-dT(*)dT(*)CGGGUACACACmUCUUCUmGG-5′.

(*) denotes phosphorothioate linkages.

The siRNA was dissolved in freshly prepared phosphate-buffered saline (PBS, pH 7.4) or 250 mM borate buffer (pH 8 or 8.5) to a concentration of 0.2 mM and stored at −20°C prior to use. BSA used was bovine serum albumin (BSA, Grade V Sigma USA). 4-[*N*-maleimidomethyl]cyclohexane-1-carboxylate (SMCC) was purchased from Pierce Chemical CO (CA, USA) and dissolved in DMSO (9.57 mM) just prior to use.

### 2.2. Reaction Conditions for Conjugation without Ultrafiltration Step

siRNA-BSA covalent conjugates were formed by reacting the 3′-amine-modified, fluorescein-labeled siRNA with the heterobifunctional linker reagent SMCC to form an irreversible amide linkage (see Figure 1). Aliquots of siRNA and SMCC linker were combined to give a ratio of 1 nmol siRNA (0.2 nM) to 40 nmol SMCC (9.58 nM) in a ~1 : 1 mixture of MilliQ water and DMSO and allowed to react for 1 h at room temperature. Excess SMCC was then removed by gel filtration chromatography using a NAP-5 column (Pharmacia, UK), with only the fluorescent flow-through collected. The quantity of siRNA collected was then determined by fluorescence using an EnVision Multilabel Plate Reader (USA) with 488 nm excitation/520 nm emission. The activated siRNA was then coupled to the surface-exposed cysteine of BSA by incubating the siRNA with 2 molar equivalents of BSA for time periods of up to 24 h (final concentrations of siRNA and BSA in the reaction mixture were 30.3 and 60.6 *μ*M, resp.). 

The components present in the reaction mixture were examined on a 10% SDS-PAGE, together with BSA and siRNA standards of known molecular weight (66.5 and 14 kDa, resp.). The gels were loaded with 1 *μ*g protein per well and run for 90 min at a constant voltage of 100 V in SDS running buffer. siRNA was visualized under UV light or using Typhoon gel scanner for detection of fluorescein emission at 520 nm, and BSA was visualised following Coomassie Brilliant Blue staining. Quantitation of the relative amounts of conjugate and unreacted siRNA was performed by densitometric analysis of siRNA and BSA bands recorded on gel scans.

### 2.3. Reaction Conditions for Conjugation with Ultrafiltration Step

Prior to conjugation, the reconstituted IGF-IR siRNA was subjected to four rounds of ultrafiltration with PBS buffer using an Amicon Ultra-4 centrifugal 3 kDa filter unit spun at 4000 rpm for 50 min at 4°C after each addition of fresh buffer. The concentrated siRNA was removed from the filter unit and quantified on an EnVision Multilabel Plate Reader (USA). Conjugation was then performed as described above.

## 3. Results and Discussion

**Figure 2:**
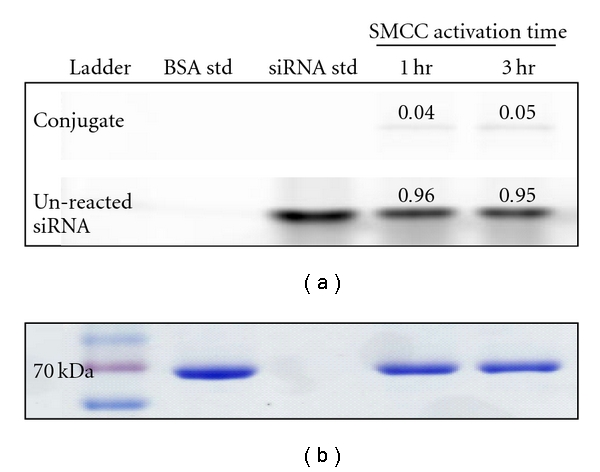
Inefficient conjugation using amine-modified siRNA without prior ultrafiltration to remove ammonium counterions. SDS-polyacrylamide gel showing outcome of siRNA-BSA conjugation reaction using 1- and 3-hour SMCC activation times. Amine-modified siRNA was activated with SMCC in a molar ratio of 40 : 1 for 1 or 3 h, followed by conjugation with BSA over 24 h. (a) Faint bands at the predicted conjugate size were observed under excitation with 488 nm light, with the majority of siRNA unreacted. (b) Only a single unconjugated albumin band was revealed by Coomassie Brilliant Blue staining in each case. siRNA std : siRNA standard; BSA std : BSA standard.

As documentation supplied with the commercially supplied amine-modified siRNA did not contain any information to suggest the contrary, it was presumed that the siRNA would be suitable for conjugation to amine-reactive compounds after simply reconstituting in an appropriate buffer. However, initial attempts to couple the siRNA to BSA via the use of SMCC, a heterobifunctional cross-linker routinely employed to link amine- to thiol-bearing molecules, met with very limited success. Following the literature precedent, siRNA was reacted (activated) with a 40-fold molar excess of SMCC prior to exposure to BSA, as described in Section 2, and at the completion of the reaction samples were analysed via PAGE. Figure 2(a) shows a representative PAGE gel, used to evaluate conjugation efficiency; scanning under 488 nm excitation reveals siRNA standard at 14 kDa in lane 3, with lanes 4 and 5 showing a significant band corresponding to unreacted siRNA band and a faint band at the size expected for the siRNA-BSA conjugate, approximately 80 KDa. Densitometric analysis indicated that only 4-5% of siRNA was conjugated to BSA and that there was no difference in yield between a one-hour and three-hour activation time. Whilst there was some batch-to-batch variability, the majority of conjugation reactions performed using several separate batches of amine-modified siRNA gave similarly poor results, with less than 20% of siRNA in conjugate form.  Figure 2(b) shows the same PAGE gel that is shown in Figure 2(a), in this case after Coomassie (protein) staining to reveal BSA. As expected, the BSA standard ran at 66 kDa. There was no clear band apparent at 80 kDa, likely indicating that the small amount of siRNA-BSA conjugate evident in Figure 2(a) was below the limit of detection using Coomassie staining.

In troubleshooting the inefficient conjugation reaction, a range of experimental parameters were varied in an attempt to drive the reaction to completion. The reaction was performed overnight, at pH values between 7 and 8.5, and using a range of siRNA, SMCC, and BSA concentrations and molar ratios. None of these strategies were successful (data not shown). Finally, we hypothesized that there may have been low-molecular-weight species in the siRNA as supplied that interfered with the conjugation reaction and, therefore, that extensive ultrafiltration or dialysis prior to activation with SMCC to remove these species might improve the outcome of the conjugation reaction. Communication with the manufacturer revealed that the siRNA was desalted using ammonium acetate; an unwise choice for amine-modified siRNA whose likely purpose is conjugation via the amine functional group, since ammonium ions would become counterions to the polyanionic siRNA. In solution, there is an equilibrium between ammonium ions and ammonia. Ammonia can act as a nucleophile, similar to the primary amine in the modified siRNA, and hence is also able to react with the succinimidyl ester group in the SMCC linker. 

**Figure 3:**
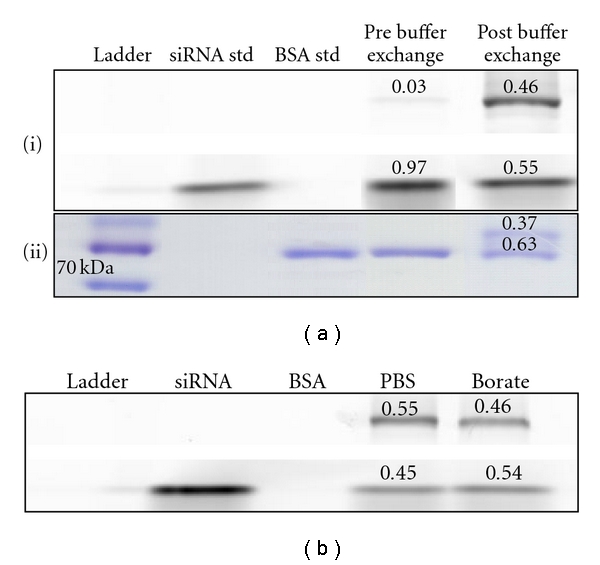
Ultrafiltration dramatically increases siRNA-albumin conjugate yield. (a) SDS-polyacrylamide gel showing outcome of siRNA-BSA conjugation reaction without prior ultrafiltration (lane 4) and subsequent to ultrafiltration of siRNA (lane 5). A 15-fold increase in conjugate band intensity was observed using ultrafiltered siRNA, compared to amine-modified siRNA simply reconstituted in buffer, when scanned at 488 nm. A visible band at the predicted conjugate size was also apparent after staining with Coomassie Brilliant Blue. (b) siRNA conjugation was efficient after buffer exchange with either PBS (pH 7.4) or borate buffer (pH 8). siRNA std : siRNA standard; BSA std : BSA standard.

Figure 3(a) (i) shows the reaction products before (lane 4) and after (lane 5) four rounds of ultrafiltration with PBS. Ultrafiltration prior to SMCC activation resulted in a 15-fold increase in the proportion of siRNA converted to the conjugate form, as evidenced by the band at 80 kDa. After Coomassie staining of this gel, a clear band at 80 kDa was evident in addition to the native BSA band, indicating the BSA component of the conjugate (Figure 3(a) (ii)). Densitometric analysis of the unreacted and conjugate BSA bands indicated that approximately one third of the BSA was involved in the reaction. This is not unexpected given that approximately 70% of albumin is in the thiol reactive (mercaptalbumin) form [11] and that a 2 : 1 albumin : siRNA ratio was employed. Thus, it appears that removal of the ammonium counterions from the amine-modified siRNA prevents the majority of the SMCC from being consumed by a competitive side reaction, and thus it allows the reaction of siRNA with SMCC to proceed more effectively.

**Figure 4:**
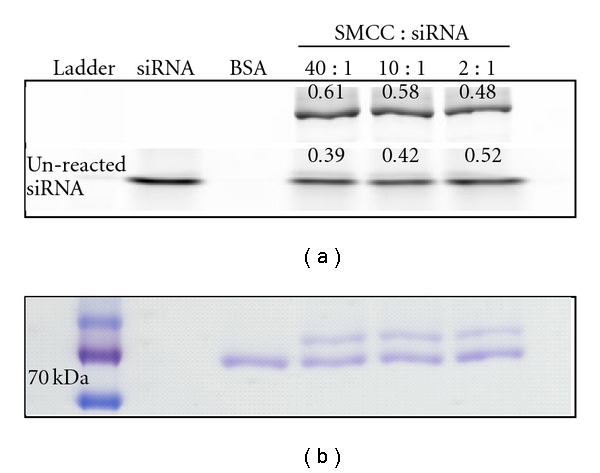
SMCC : siRNA ratio can be reduced without loss of yield after ultrafiltration of amine-modified siRNA. Conjugation of amine-modified siRNA with BSA using 40 : 1, 10 : 1, and 2 : 1 SMCC-to-siRNA molar ratios. Conjugates formation was observed for each of the SMCC : siRNA ratios; reducing the ratio from 40 : 1 to 2 : 1 had relatively little effect on conjugate yield. siRNA std : siRNA standard; BSA std : BSA standard.

 Having demonstrated that the reaction efficiency was dramatically improved by ultrafiltration using PBS, we anticipated that the ratio of SMCC linker to siRNA used (40 : 1) was unnecessarily high.  Figure 4 shows evaluation of the effect of reducing the SMCC : siRNA ratio on the outcome of the conjugation reaction. There was no reduction in the proportion of siRNA in conjugate form upon reducing the SMCC : siRNA ratio from 40 : 1 to 10 : 1, and only a modest decrease occurred when the ratio was further reduced to 2 : 1 (48% of siRNA in conjugate form versus 61% for 40 : 1 ratio, Figure 4(a)). Thus, it was possible to reduce the SMCC concentration during the reaction, with the advantage of reducing the amount of excess linker remaining after the reaction, as well as lowering the cost of synthesis.

## 4. Conclusions

These data clearly demonstrate that siRNA supplied commercially with terminal amine groups can require ultrafiltration or dialysis prior to exposure to linker reagents in order to remove ammonium ions remaining after the desalting step used in the purification process. Researchers planning to perform conjugation experiments should consult with the supplier as to the purification conditions, and if it is known or suspected that ammonium-containing salts were used, an ultrafiltration step, as described herein, should be performed prior to use of the siRNA.
